# Post-stroke depression, anxiety and apathy: 12-month trajectories, predictors and treatment effects

**DOI:** 10.1192/j.eurpsy.2026.10723

**Published:** 2026-07-31

**Authors:** B. Mihaela, D. Jelaga

**Affiliations:** Department of Mental Health, Medical Psychology and Psychotherapy, Nicolae Testemițanu State University of Medicine and Pharmacy, Chisinau, Moldova, Republic of

## Abstract

**Introduction:**

Post-stroke depression (PSD), anxiety (PSA) and apathy are common and associated with poorer functional recovery and higher mortality. Recent evidence clarifies first-year trajectories and treatment effects, yet estimates vary across instruments and settings.

**Objectives:**

(1) Quantify 3/6/12-month prevalence and course of PSD, PSA and apathy; (2) identify independent 12-month predictors; (3) estimate absolute effects and safety of pharmacological and psychological interventions.

**Methods:**

Databases: MEDLINE/PubMed, Embase, PsycINFO, Web of Science (2020–2025). Eligibility: adults with ischaemic/haemorrhagic stroke; longitudinal cohorts/registries, meta-analyses/IPD/meta-reviews, RCTs; validated 3–12-month outcomes. Exclusions: paediatric, case reports, non-validated tools, mixed neurological cohorts without stroke-specific data. Flow: 1,186 records; 864 after deduplication; 112 full texts; 17 included; 95 excluded. Populations: mean age 58–70 years; 38–50% women; inpatient/outpatient/community. Dual screening; narrative risk-of-bias (RoB2/ROBINS-I). Outcomes: prevalence/incidence, persistence/remission (pp), subgroup differences, absolute effects (ARR, NNT/NNH), adverse events.

**Results:**

PSD: pooled 27% (interview 24%, scales 29%); among ≤3-month cases, 53% persisted to 12 months and 44% remitted; new-onset 3–12 months 9%; 71% of annual episodes began ≤3 months.

PSA: 20–25% in year 1; at 3–6 months some cohorts 35%, higher in women (44.5% vs 28.5% in ischaemic stroke); ≈24% at 12 months.

Apathy: 33.0% (95% CI 27.6–38.4); longitudinal 7% persistent-high, 33% moderate, 50% low/none; self-reported apathy rose: AES 10.8%→22.4% (+11.6 pp); DAS 3.6%→11.0% (+7.4 pp) by 12 months.

Predictors (12 months): higher baseline NIHSS/mRS, cognitive impairment, prior affective disorder, low social support; sex differences mainly ischaemic (not consistent in haemorrhagic).

Interventions: fluoxetine 20 mg for 6 months vs placebo reduced new depression at 6 months 13.42%→10.05% (ARR 3.37%; NNT=30) with no functional benefit, but increased adverse events—fractures 1.39%→3.15% (NNH=57), injurious falls 4.51%→6.26% (NNH=57), seizures 1.80%→2.64% (NNH=119), hyponatraemia 0.61%→1.22% (NNH=164). Psychological therapies (CBT/behavioural activation), national routine-care dataset (n=7,597): 71.3% reliable improvement, 49.2% reliable recovery (associative, non-randomised), better when initiated ≤6 months. Adherence/discontinuation not consistently reported.

**Conclusions:**

Within 12 months post-stroke, PSD ≈27%, PSA 20–25% and apathy ≈33%. Depression starts early and persists in ~1/2 early cases; apathy is comparatively stable and less treatment-responsive. Stepwise screening at 0–3–6–12 months, stratification by neurological severity/cognition and proactive social-support enhancement are warranted. SSRIs: small preventive benefit with safety trade-offs; psychological therapies: meaningful gains—integrate early.

**Disclosure of Interest:**

None Declared

